# Statistical learning methods for improving predictive performance in time-dependent survival models

**DOI:** 10.1186/s44342-025-00050-7

**Published:** 2025-09-01

**Authors:** Hyungwoo Seo, Wonil Chung

**Affiliations:** 1 Department of Statistics and Actuarial Science, Soongsil University, Seoul, 06978 South Korea; 2 Program in Genetic Epidemiology and Statistical Genetics, Harvard T.H. Chan School of Public Health, Boston, MA 02115 USA

**Keywords:** Time-dependent survival models, Cox proportional hazards models, Random survival forest, DeepSurv, DeepHit, COVID-19

## Abstract

**Background:**

The COVID-19 pandemic has highlighted the need for survival models to assess risk factors and time-dependent effects in infectious diseases. However, the Cox proportional hazards (PH) model, which assumes constant covariate effects, struggles to capture disease dynamics. This underscores the need for advanced models that incorporate time-dependent coefficients and covariates for improved accuracy.

**Methods:**

To address the need for modeling time-dependent effects and covariates, we applied a stratified Cox PH model with multiple time intervals to better satisfy the PH assumption. We conducted simulations to evaluate the performance of machine learning and deep learning survival models, including random survival forest (RSF), DeepSurv, and DeepHit. To improve time-dependent effect estimation, we introduced a refined time-interval division and a weighted sum approach for integrated hazard ratios of COVID-19 variants. The event of interest was death, and the specific risk compared was the risk of death from the start of the study to either death or the last follow-up among infected versus uninfected individuals.

**Results:**

Our results showed that increasing the number of time intervals improved predictive accuracy. When the PH assumption held, the Cox PH model outperformed machine learning and deep learning models. Applying our approach to UK Biobank data, expanding time intervals from five to fifteen enhanced performance. The previously reported hazard ratio of 7.333 for the pre-Delta period was refined to 29.359 for the Early variant, 20.734 for EU1, and 4.079 for Alpha, revealing a decline in risk across variants.

**Conclusions:**

These findings suggest that refining time intervals improves the understanding of time-dependent effects in infectious diseases. Incorporating stratified intervals and advanced models enhances risk assessment and predictive accuracy for COVID-19 and other evolving diseases.

**Supplementary Information:**

The online version contains supplementary material available at 10.1186/s44342-025-00050-7.

## Introduction

During the COVID-19 pandemic, survival models were widely used to analyze death-related covariates and identify risk factors affecting survival time—both of which were crucial for developing effective disease management and treatment strategies [1–3]. These models provided valuable insights into the progression of the disease and the factors influencing patient outcomes. In the post-COVID-19 era, survival models remain essential for studying risk factors associated with various infectious diseases, including COVID-19. By capturing disease progression and mortality dynamics, they help researchers and healthcare professionals assess the impact of different variables on patient survival. Furthermore, studies on other infectious diseases, such as Ebola virus and human metapneumovirus, have further demonstrated the effectiveness of this approach [4, 5].

In survival modeling for infectious diseases, two main approaches are considered. The first defines survival time as the number of days from infection to either death or the last follow-up date. The second defines it as the number of days from the start of the study to either the date of death or the last follow-up. The first approach offers a relatively straightforward modeling process, illustrating how environmental factors influence survival in infected individuals. In contrast, the second approach is more complex, as infections occur continuously throughout the study period, and their effects typically decline over time [6–8]. This complexity requires accounting for two distinct time-varying aspects: the hazard ratio associated with infection may change over time (time-dependent coefficients), and infection status itself changes during follow-up as individuals transition from uninfected to infected (time-dependent covariates). This complexity necessitates survival models that incorporate both time-dependent coefficients and time-dependent covariates. To address this, the analysis period is divided into smaller intervals that satisfy the proportional hazards assumption, and infection is treated as a time-dependent covariate that activates at the start of the interval in which infection occurs. This approach enables the estimation of variant-specific effect sizes aligned with periods of variant predominance while maintaining model interpretability.

Our previous study employed Cox proportional hazards (PH) models stratified by time intervals to assess the impact of SARS-CoV-2 infections, type 2 diabetes (T2D), and its genetic predisposition over the study period [9]. The results indicated that mortality risk was highest during the Early variant phase and lowest with the Omicron variants, and both T2D and its genetic predisposition were significantly associated with the severity and mortality of COVID-19. However, to meet the PH assumption, the study treated the period before the emergence of the Delta variant as a single, homogeneous interval, which prevented distinguishing between temporal variations in risk for specific variants such as the Early and Alpha phases. Furthermore, we did not explore alternative statistical learning models beyond the stratified Cox PH models, potentially limiting the scope of our analysis.

In this study, we propose a stratified Cox PH model that divides the study period into smaller time intervals, allowing for a more precise estimation of time-dependent covariate effects. Additionally, we introduce a weighted sum approach that aggregates multiple time intervals to derive integrated hazard ratios for different COVID-19 variants. We further compare the predictive performance of Cox PH models with machine learning and deep learning survival models, including the random survival forest (RSF) [10], DeepSurv [11], and DeepHit [12], through simulation studies under various parameter settings. Moreover, we assess how event rates and interval censoring rates that we define as censoring occurring during the study period, excluding right censoring at the end of follow-up, influence model performance in scenarios with and without time-dependent effects. Finally, we apply our methods to real SARS-CoV-2 infection data from the UK Biobank, evaluating predictive performance when incorporating finer time intervals with various statistical learning methods.

## Methods

### Study population and design

The UK Biobank, a large-scale cohort study, enrolled 503,325 participants aged 40 to 69 from 22 research centers across the UK between 2006 and 2010 [13]. For this analysis, we selected 430,747 individuals of European ancestry based on the availability of high-quality genotype data and comprehensive phenotype and covariate information. We utilized self-reported questionnaire data, hospital inpatient records, and death registry data, all updated as of December 19, 2022. The SARS-CoV-2 infection phenotype included 100,827 cases and 329,920 controls [14]. Participants diagnosed with COVID-19 were classified into seven variant groups: Early variant, EU1 (20E), Alpha (20I), Delta (21J), Omicron (21K), Omicron2 (21L), and Omicron3 (22B). The Early variant category encompassed all strains identified before the emergence of the EU1 variant. For genetic analysis, single nucleotide polymorphisms (SNPs) were filtered out if they showed deviation from Hardy–Weinberg Equilibrium (HWE) (*P* < 1 × 10⁻^12^), had per-variant missing call rates > 10%, per-sample missing rates > 10%, a minor allele frequency (MAF) < 0.01, or an imputation quality score (INFO) < 0.8, resulting in 9,572,556 SNPs retained for analysis.

### Polygenic risk score (PRS)

The polygenic risk score (PRS) quantifies an individual’s genetic predisposition to a specific trait by multiplying the genotype dosage of each variant by its corresponding weight and summing across all variants [15, 16]. To compute T2D PRS for 430,747 participants, we employed a ten-fold cross-validation (CV) approach, where samples were randomly divided into ten folds, with each fold serving as a validation set while the remaining nine formed the training set. First, genome-wide association study (GWAS) summary statistics were generated from the ten training sets for each trait. The PRS was then computed for the corresponding validation sets using the LDpred method [17]. This process yielded PRS scores for all individuals. To minimize bias, PRS scores from the ten folds were standardized before integration. Candidate PRSs were generated across a range of tuning parameters, specifically the proportion of causal variants. The final PRS was selected based on the highest discriminative performance, determined by the maximum area under the curve (AUC).

### Survival models

For survival analysis, we employed four models: the stratified Cox PH model, RSF, DeepSurv, and DeepHit. The stratified Cox PH model accounts for variations across different strata, allowing each time interval to have a distinct reference hazard function [9]. RSF constructs multiple survival trees by drawing bootstrap samples from the original dataset and estimates the cumulative hazard function by aggregating individual tree results [10]. DeepSurv, a deep feed-forward neural network, models the impact of covariates on an individual’s hazard rate, with network weights parameterizing the hazard function. It is trained by minimizing the average negative log partial likelihood with an ${{\ell}}_{2}$ regularization (Ridge) term to prevent overfitting. DeepSurv utilizes gradient descent for optimization, employing scaled exponential linear units (SELU) [18] as the activation function and adaptive moment estimation (Adam) [19] for efficient convergence. Learning rate scheduling dynamically adjusts the learning rate during training to enhance performance [20]. DeepHit, another deep feed-forward neural network, predicts the effects of covariates on hazard rates and is particularly suited for competing risks scenarios [12]. Its loss function consists of two components: ${\mathcal{L}}_{\text{total}}={\mathcal{L}}_{1}+{\mathcal{L}}_{2}$. Here, ${\mathcal{L}}_{1}$ represents the log-likelihood of the joint distribution of the first hitting time and event, while ${\mathcal{L}}_{2}$ incorporates cause-specific ranking loss functions to improve predictive accuracy. More details on RSF, DeepSurv, and DeepHit models are provided in Supplementary notes 1–3. All four models use the same approach of dividing the analysis period into time intervals to enable the estimation of time-varying effects within those intervals. The stratified Cox PH model is a traditional parametric model that assumes proportional hazards within each interval. RSF is a nonparametric, machine learning–based model that can flexibly capture complex, non-linear effects. DeepSurv and DeepHit are deep-learning–based models with many parameters, allowing them to model complex time-varying effects even when the PH assumption does not hold. This flexibility can lead to better estimation of time-varying effects and improved predictive performance, as reflected in measures such as the time-dependent C-index.

### Time-dependent C-index

We used the time-dependent concordance index (C-index) to evaluate the predictive performance for time-to-event outcomes [21, 22]. The time-dependent C-index ($${C}^{td})$$ measures a model’s ability to accurately rank survival probabilities across different time points while accounting for time-dependent risks. To define this metric, we introduce basic notation with discrete time points. For $$k$$ th time point $${t}_{(k)}$$, $${D}_{i}\left({t}_{\left(k\right)}\right)=1$$ indicates that the subject $$i$$ has experienced the event (diseased) at $${t}_{(k)}$$ and $${D}_{i}\left({t}_{\left(k\right)}\right)=0$$ indicates that the subject $$i$$ remains event-free up to $${t}_{(k)}$$. The survival probability at $${t}_{(k)}$$ is denoted as $$S\left({t}_{(k)}|{{\varvec{X}}}_{{\varvec{i}}}\left({\varvec{t}}\right)\right)$$ where $${{\varvec{X}}}_{{\varvec{i}}}\left({\varvec{t}}\right)$$ represents the vector of potentially time-dependent covariates of subject $$i$$. The probability that a pair of subjects $$(i,j)$$ are comparable $$({\pi }_{\text{comp}})$$ and the probability that they are in time-dependent concordance ($${\pi }_{conc}^{td})$$ are defined as $${\pi }_{\text{comp}}={\sum }_{k=0}^{K}\text{Pr}\left({D}_{i}\left({t}_{\left(k\right)}\right)=1 \&{ D}_{j}\left({t}_{\left(k\right)}\right)=0\right)$$, $${\pi }_{conc}^{td}={\sum }_{k=0}^{K}\text{AUC}\left({t}_{(k)}\right)\cdot \text{Pr}\left({D}_{i}\left({t}_{\left(k\right)}\right)=1 \&{ D}_{j}\left({t}_{\left(k\right)}\right)=0\right)$$ where $$AUC\left(t_{\left(k\right)}\right)=Pr\left(S\left(t_{\left(k\right)}\vert{\boldsymbol X}_{\boldsymbol i}\left(\boldsymbol t\right)\right)<S\left(t_{\left(k\right)}\vert{\boldsymbol X}_{\boldsymbol j}\left(\boldsymbol t\right)\right)\vert D_i\left(t_{\left(k\right)}\right)=1\&D_j\left(t_{\left(k\right)}\right)=0\right)$$. The time-dependent C-index is then computed as $$C^{td}=\frac{\mathrm\pi_{\mathrm{conc}}^{\mathrm{td}}}{{\mathrm\pi}_{\mathrm{comp}}}Pr\left(S\left(T_i\vert{\boldsymbol X}_{\boldsymbol i}\left(\boldsymbol t\right)\right)<S\left(T_i\vert{\boldsymbol X}_{\boldsymbol j}\left(\boldsymbol t\right)\right)\vert T_i<T_j\&D_i=1\right)$$. The time-dependent C-index is evaluated at each observed time point where an event or censoring occurs, allowing for dynamic tracking of risk changes over time. This makes it well-suited for both proportional and non-proportional hazards models.

### Simulation under the PH assumption

To compare the predictive performance of survival models under the PH assumption, we conducted simulations using SARS-CoV-2 infected individuals from the UK Biobank [14]. We randomly sampled 10,000 individuals twice to create independent training and testing datasets. For each dataset, we simulated survival times and events using the following procedure. The survival times of each individual were simulated using the hazard function as $h\left(t\right)=\gamma \lambda {t}^{\gamma -1}\mathit{exp}\left({\varvec{\beta}}{{\varvec{C}}}_{{\varvec{i}}}\right)$ [23]. Here, $\gamma$, $\lambda$ are the shape and scale parameters of the Weibull baseline hazard, ${{\varvec{C}}}_{{\varvec{i}}}$ is the set of clinical covariates such as age, sex, body mass index (BMI), T2D or T2D PRS groups, genotype array, and COVID-19 variants and ${\varvec{\beta}}$ is the corresponding effects. We considered two event rate settings: 5% and 10%. For each event rate setting, we additionally applied two separate interval censoring scenarios with rates of 0% and 5%, resulting in four combinations in total. For each set of parameters, we performed a total of 100 simulations. The censoring mechanism was designed to be independent of event times, with censoring status generated randomly and censoring times randomly selected within the range of simulated survival times.

### Simulation with time-dependent effects

We conducted simulations to assess the predictive performance of survival models incorporating time-dependent effects. The survival times of each individual were simulated using the hazard function as $h\left(t\right)=\gamma \lambda {t}^{\gamma -1}\mathit{exp}\left({\beta }_{1}{X}_{i}+{\beta }_{2}{X}_{i}\times \text{log}(t+10)+{{\varvec{\beta}}}_{3}{{\varvec{C}}}_{{\varvec{i}}}\right)$ where ${X}_{i}$ is the time-dependent covariate for subject $i$ representing the indicator variable for SARS-CoV-2 infection. In this model, ${\beta }_{1}$ denotes the log hazard ratio for SARS-CoV-2 infection while ${\beta }_{2}$ quantifies how the effect of infection status on the log hazard ratio changes over time through its interaction with the log-transformed time [23]. The time-dependent effect is modeled as an interaction between the infection status indicator and the log-transformed time. The effect sizes of each covariate were set to values similar to those estimated when fitting the same model to real data. The simulation considered two infection rates, 25% and 50%, and two event rates, 5% and 10%. For both the training and test datasets, survival times and event occurrences were generated through a structured process. First, survival times were simulated using the predefined hazard function. Next, two scenarios were established based on event rates of 5% and 10%. Within each scenario, two additional conditions were examined: one with 0% interval censoring and another with 5% interval censoring. By combining these conditions with the infection rates of 25% and 50%, a total of eight distinct datasets were generated. Each parameter configuration was simulated 100 times. To incorporate time-dependent effects, the generated datasets were further divided into 5 and 10 time-intervals using the survSplit function in R. In the case of five intervals, survival times were segmented into 200-day periods: $\left(\text{0,200}\right]$, $\left(\text{200,400}\right]$, $\left(\text{400,600}\right]$, $\left(\text{600,800}\right]$, and $(800,\infty )$. In the case of ten intervals, survival times were divided into 100-day periods. Within each interval, baseline covariates remained unchanged, and SARS-CoV-2 infection status was defined based on whether an individual had been infected at least once before the end of the respective time interval. Details of the simulation settings are summarized in Table 1.

**Table 1:** Simulation settings for non-time-dependent (PH assumption) and time-dependent effects

Model type	Parameter	Setting	Models for analysis
Non-time-dependent effects	Event rate	5%, 10%	Cox PH, RSF, DeepSurv, DeepHit
	Interval censoring rate	0%, 5%	
Time-dependent effects	Event rate	5%, 10%	Stratified Cox PH, RSF, DeepSurv, DeepHit
	Interval censoring rate	0%, 5%	
	SARS-CoV-2 infection rate	25%, 50%	
	Number of time intervals	0, 5, 10	

### Survival analysis with real data

We first evaluated the predictive performance of survival models using all participants (*N* = 430,747) from the UK Biobank. Survival time was defined as the number of days from the start of the study to either the date of death or the last follow-up. Our previous study utilized a model with five time-intervals [9], whereas the new model assigned each COVID-19 variant to distinct time-intervals. The five-interval model was defined based on the emergence and predominance of COVID-19 variants, using surveillance data to identify when each variant surpassed 95% prevalence in the population (e.g., pre-Delta, Delta, Omicron1, Omicron2, Omicron3). To ensure compliance with the PH assumption, we evaluated each interval using a Cox PH model with the Schoenfeld residuals test, and any intervals that violated this assumption were further divided into two equal-length intervals. This process was repeated iteratively until all intervals satisfied the PH assumption, resulting in a total of fifteen time-intervals. Furthermore, the hazard ratios estimated from the fifteen intervals were aggregated using a weighted sum approach to produce integrated hazard ratio estimates and standard deviations for each of the seven COVID-19 variants. For the model with five-time intervals, the dataset was divided into five groups using four cutoff points: 494, 672, 757, and 851. In contrast, the model with fifteen time-intervals partitioned the dataset into fifteen groups using fourteen cutoff points: 111, 166, 194, 222, 284, 346, 364, 383, 402, 420, 494, 672, 757, and 851. The predictive performance of the survival models was assessed using a tenfold CV approach.

We also conducted survival analysis exclusively on individuals infected with SARS-CoV-2. Here, survival time was defined as the number of days from SARS-CoV-2 infection to either death or the last follow-up. Covariate adjustments were made for factors including age, sex, BMI, T2D or T2D PRS groups, the top four genotype principal components (PCs), and the genotyping array. An additional model further incorporated COVID-19 variants as an additional covariate.

## Results

### Overview of the study

**Fig. 1:**
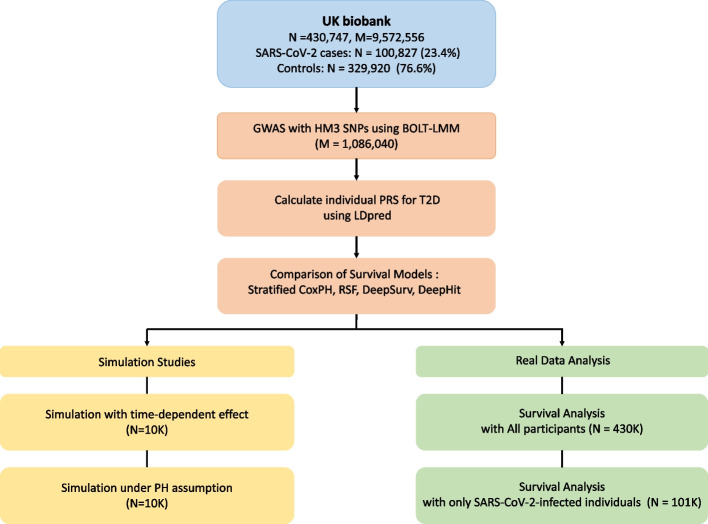
Overview of the study. We analyzed 430,747 individuals from the UK Biobank to investigate survival outcomes related to SARS-CoV-2 infection. GWAS were conducted using BOLT-LMM, and PRS were computed using LDpred. Survival models, including stratified Cox PH, RSF, DeepSurv, and DeepHit, were compared to assess survival risks over time. The analysis incorporated both simulations and real data. Simulations were conducted to evaluate time-dependent effects and assess the PH assumption, each with 10,000 simulated cases. Survival analysis was performed on both the full cohort and a subset of 100,827 SARS-CoV-2-infected individuals

The study overview is illustrated in Fig. 1. This study investigates survival outcomes related to SARS-CoV-2 infection using data from 430,747 individuals in the UK Biobank. Various survival models, including stratified Cox PH, RSF, DeepSurv, and DeepHit, were applied to assess survival risks over time. Our analysis consisted of both simulation studies and real data analysis. For simulation studies, survival outcomes were modeled under different PH assumptions to examine time-dependent effects and the validity of the PH assumption, each with 10,000 simulated cases. These simulations helped capture dynamic changes in hazard ratios over time and improve survival estimation. In the real data analysis, survival models were applied to the full cohort (*N* = 430,747) and a subset of SARS-CoV-2-infected individuals (*N* = 100,827) to evaluate the impact of SARS-CoV-2 infection on survival outcomes. The event of interest was death from the start of the study to either death or the last follow-up, comparing the risk between individuals who became infected and those who remained uninfected, with uninfected serving as the reference group. Additionally, GWAS for T2D were performed using BOLT-LMM and T2D PRS was calculated using LDpred. By integrating simulated and real data, this study systematically evaluates survival modeling approaches and their effectiveness in capturing the time-dependent effects of SARS-CoV-2 infection.

### Simulation results under the PH assumption

**Fig. 2:**
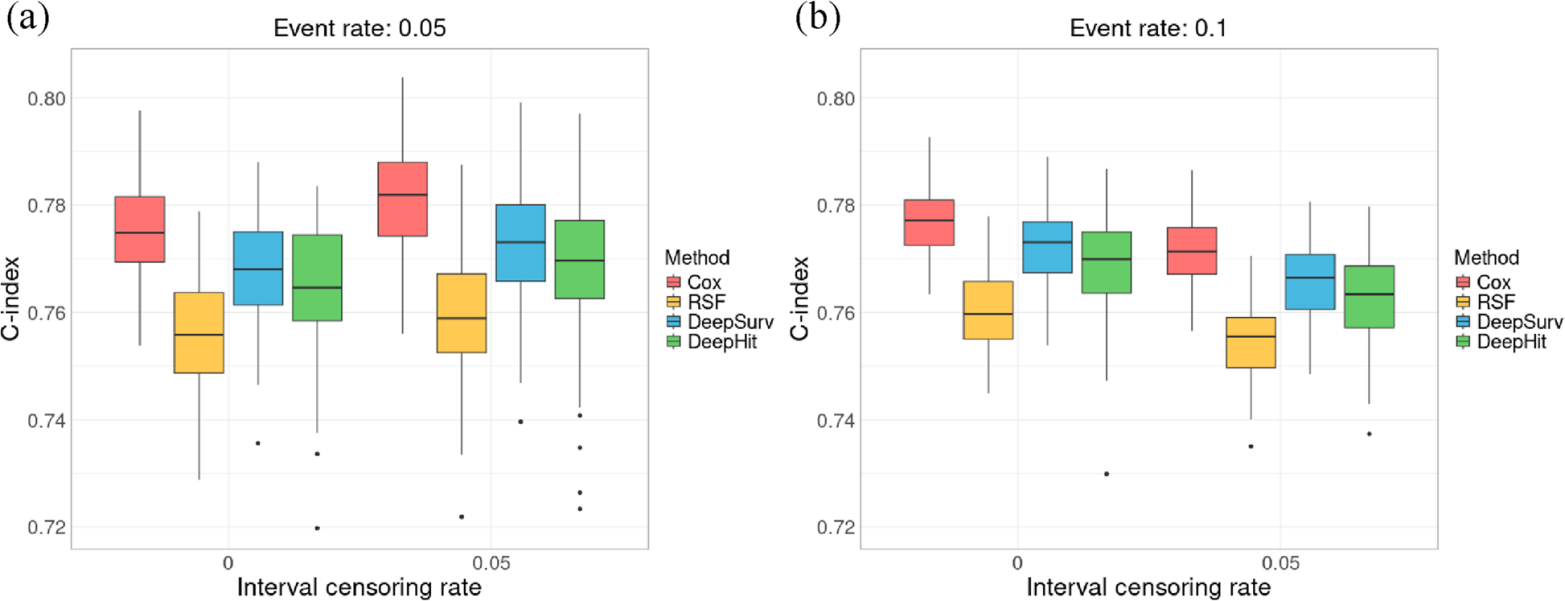
Boxplots for simulation results under the PHassumption.** a** C-index comparison across different survival models in simulations with a 5% event rate and interval censoring rates of 0% and 5%; (b) C-index comparison across different survival models in simulations with a 10% event rate and interval censoring rates of 0% and 5%

When evaluating the C-index across different survival models in simulations under the PH assumption, the Cox PH model consistently demonstrated the highest predictive performance across all scenarios, while the RSF model exhibited the lowest performance, as shown in Fig. 2. The deep learning-based models, DeepSurv and DeepHit, showed comparable predictive accuracy, consistently outperforming RSF model but not surpassing the predictive ability of the Cox PH model. The results of the PH assumption test for the Cox PH model, presented in Supplementary Table S1, confirm that the assumption was satisfied in these simulations. This validation supports the reliability of the Cox PH model in scenarios where the PH assumption holds. The impact of interval censoring varied depending on the event rate. When the event rate was 5%, applying 5% interval censoring slightly increased the C-index across all survival models compared to no interval censoring, as it reduced the number of comparable pairs, as illustrated in Fig. 2a. However, when the event rate was 10%, interval censoring decreased the C-index across all models, likely due to the loss of survival information caused by a higher probability of event occurrence, as shown in Fig. 2b. All results are provided in Supplementary Table S2.

### Simulation results with time-dependent effects

**Fig. 3:**
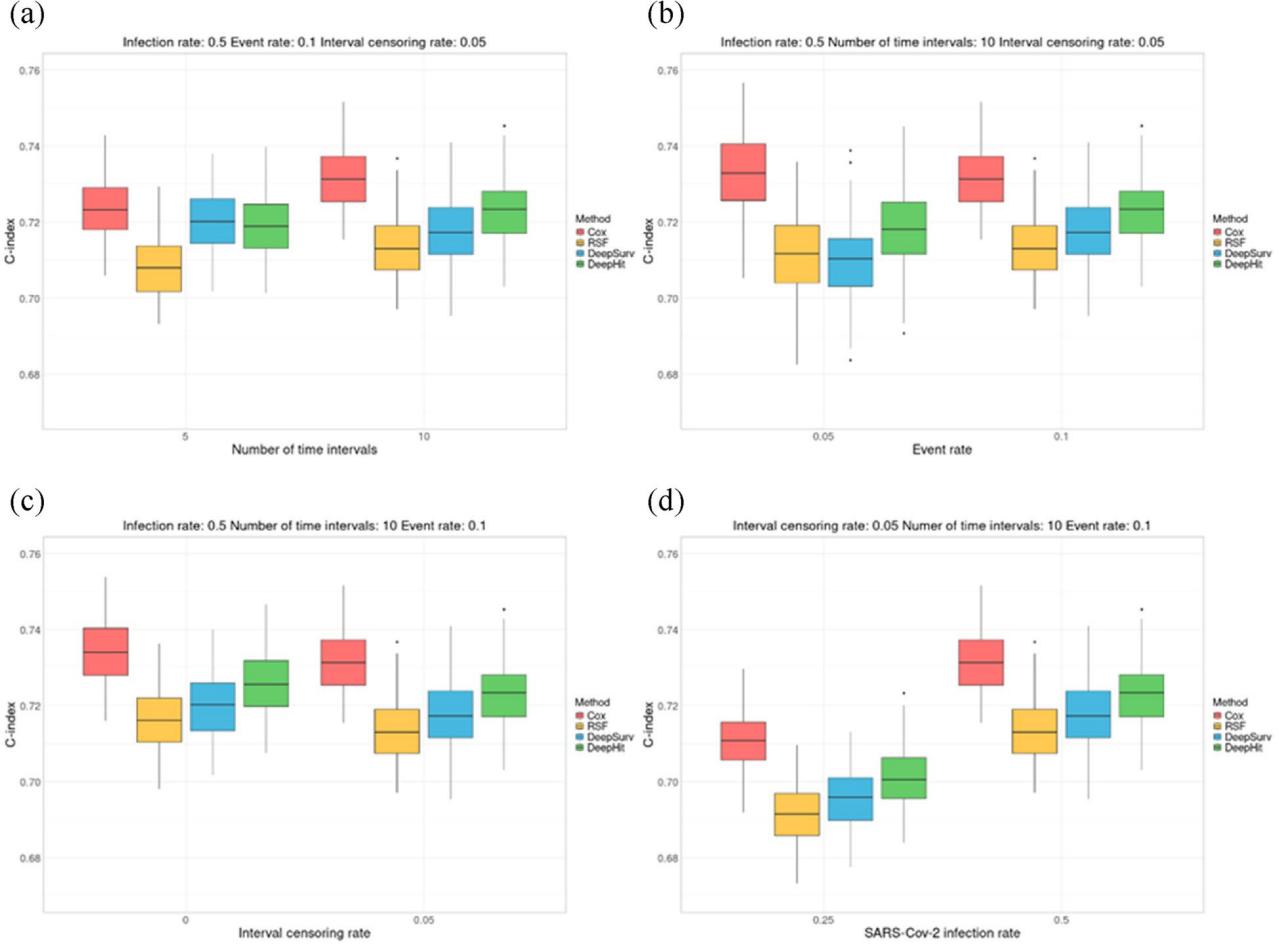
Boxplots for simulation results with time-dependent effects.** a** Time-dependent C-index comparison across different survival models in simulations with a 10% event rate, 50% SARS-CoV-2 infection rate, no interval censoring, and five or ten time intervals. **b** Time-dependent C-index comparison across different survival models in simulations with ten time intervals, a 50% SARS-CoV-2 infection rate, 5% interval censoring, and event rates of 5% and 10%. **c** Time-dependent C-index comparison across different survival models in simulations with a 10% event rate, a 50% SARS-CoV-2 infection rate, ten time intervals, and interval censoring rates of 0% and 5%. **d** Time-dependent C-index comparison across different survival models in simulations with a 10% event rate, a 5% interval censoring rate, ten time intervals, and SARS-CoV-2 infection rates of 25% and 50%

To evaluate the impact of the number of time-intervals on predictive performance, we trained RSF, DeepSurv, and DeepHit models using three different time-interval configurations: one without time-interval splitting, another with five-time intervals, and a third with ten time-intervals. The time-dependent C-index was used to compare predictive performance across different survival models. Supplementary Table S3 presents the results of the PH assumption test for the Cox PH model, indicating that the assumption was violated when time intervals were not divided. As a result, the Cox PH model was only evaluated under two conditions: one with five time-intervals and another with ten time-intervals. Figure 3 illustrates boxplots showing variations in predictive performance across different conditions, using a baseline configuration of a 10% event rate, a 50% SARS-CoV-2 infection rate, 5% interval censoring, and 10 time-intervals. The Cox PH model consistently demonstrated the highest predictive accuracy, consistent with findings from the PH-assumed simulations, while the ranking of the other survival models remained unchanged. Increasing the number of time intervals from five to ten generally improved performance, as shown in Fig. 3a. When the event rate decreased from 10 to 5%, predictive accuracy slightly declined or showed increased variance, as observed in Fig. 3b. Models trained without interval censoring generally outperformed those with 5% interval censoring, as shown in Fig. 3c. The most significant decline in predictive performance was observed when the SARS-CoV-2 infection rate decreased from 50 to 25%, suggesting that reducing information on the most influential variable in the model led to a notable reduction in predictive accuracy, as shown in Fig. 3d. Boxplots of the C-index for all simulation results are presented in Supplementary Figs. S1-S4 and Supplementary Table S4.

### Survival analysis results

**Fig. 4:**
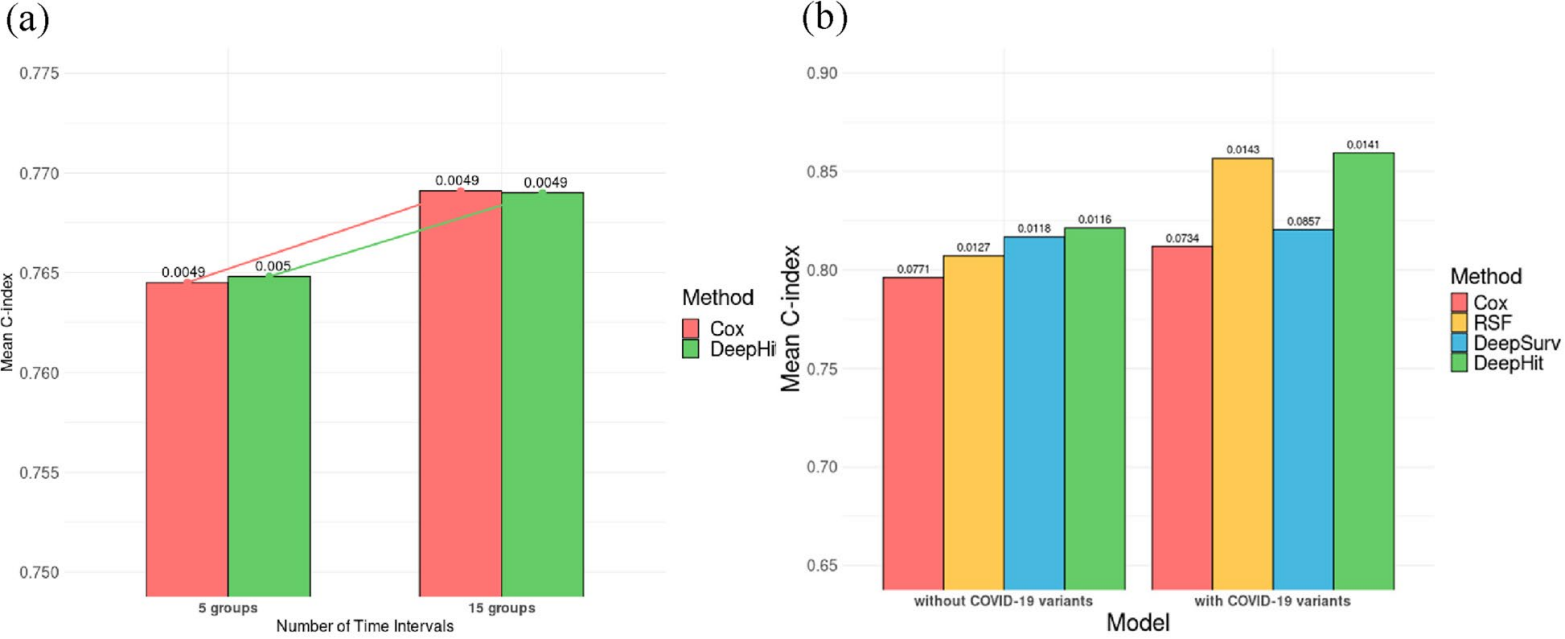
Average C-Index based on ten-fold CV for real data analyses.** a** Average C-index for survival models including T2D among all participants, stratified by the number of time intervals. **b** Average C-index for survival models including T2D among SARS-CoV-2-infected individuals, comparing models with and without COVID-19 variants

Figure 4a presents bar plots of the average C-index based on ten-fold CV for survival models including T2D with all participants. The predictive performance of the stratified Cox PH model and DeepHit improved as the number of time intervals increased, suggesting that a higher number of time intervals enhances the model’s ability to capture the time-dependent effect of SARS-CoV-2 infection more accurately at each interval. The results of the PH assumption tests for the stratified Cox PH models, presented in Supplementary Tables S5 and S6, confirm that the assumption is well satisfied in this analysis. All results are provided in Supplementary Table S7.

Figure 4b presents bar plots of the average C-index based on ten-fold CV for survival models including T2D with only SARS-CoV-2-infected individuals. The Cox PH model exhibited lower predictive performance compared to non-PH-assumed models such as RSF, DeepSurv, and DeepHit, likely due to the violation of the PH assumption, as indicated in Supplementary Table S8. Incorporating COVID-19 variants into the model improved overall predictive performance, suggesting that different COVID-19 variants are associated with varying levels of hazard. All results are summarized in Supplementary Table S9.

### Estimation of hazards ratios for COVID-19 variants

**Fig. 5:**
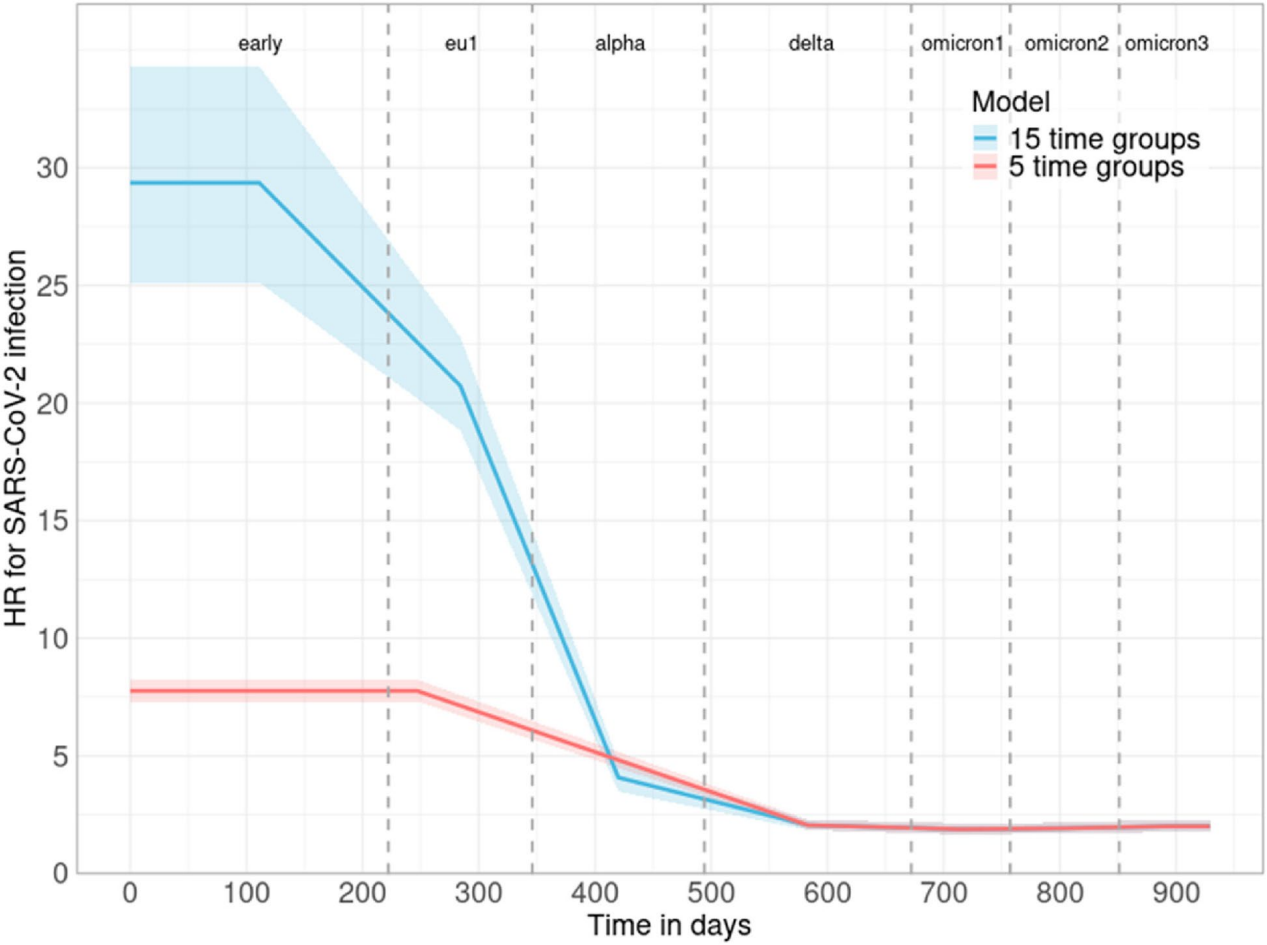
Hazard ratio for SARS-CoV-2 infection by COVID-19 variant across different time intervals. The estimated hazard ratios for each time group are connected to form lines representing two models: the blue line for the 15 time group model and the red line for the 5 time group model. The shaded area around the blue line represents the 95% confidence intervals for the 15 time group model estimates, while the shaded area around the red line represents the 95% confidence intervals for the 5 time group model estimates. This figure illustrates how increasing the number of time intervals in a stratified Cox PH model influences the estimation of the time-dependent effects of SARS-CoV-2 infection

To improve the estimation of time-dependent coefficients and the effects of COVID-19 variants, we propose a novel approach that increases the number of time intervals in stratified Cox PH models. By introducing 15 time intervals, each COVID-19 variant spans at least one interval, allowing for a more refined estimation of its effect over time. To estimate the hazard ratio for each of the seven COVID-19 variants, we aggregate the effects of SARS-CoV-2 infection across multiple time intervals using a weighted sum approach, where the weight is determined by the length of each interval. Specifically, the effect of SARS-CoV-2 infection for each COVID-19 variant is computed by multiplying the effect of SARS-CoV-2 infection in each time interval by its respective weight, yielding an integrated value. The weight is defined as the proportion of the interval’s length relative to the total duration of the COVID-19 variant covering that interval. Using this weighted sum approach, the 15 hazard ratio estimates and their corresponding standard errors (SE) from the 15 time intervals are aggregated for each COVID-19 variant. The variance of the weighted sum was calculated analytically using the standard formula for a weighted sum of independent estimates, and the final SE was obtained as the square root of this variance, resulting in seven final hazard ratio estimates and SEs. The hazard ratios and *P*-values for SARS-CoV-2 infection based on the stratified Cox PH model with 15 time intervals are presented in Supplementary Table S10. Compared to results obtained using five time intervals, the hazard ratio of SARS-CoV-2 infection, originally estimated as 7.333 in the pre-delta period (Supplementary Table S11), is now estimated at 29.359 for the Early variant, 20.734 for the EU1 variant, and 4.079 for the alpha variant. As illustrated in Fig. 5, increasing the number of time intervals enhances the accuracy of representing the time-dependent effects of SARS-CoV-2 infection. By dividing time intervals into smaller, more discrete segments, the model more effectively captures dynamic changes in hazard ratios over time. This approach not only improves the overall model fit but also provides a clearer understanding of the effect of SARS-CoV-2 infection at specific time points.

## Discussion

In this study, we addressed the challenge of fitting survival models that incorporate both time-dependent coefficients and time-dependent covariates. To overcome this issue, we employed a stratified Cox PH model with a strategy that partitions time into discrete intervals. By increasing the number of time intervals, we aimed to enhance the estimation of time-dependent effects and more accurately capture the impact of COVID-19 variants. To quantify the hazard ratio for each of the seven SARS-CoV-2 variants, we introduced a weighted sum approach, which consolidates the effect of SARS-CoV-2 infection across multiple time intervals into a single representative value. Additionally, we conducted simulations under various conditions to compare the predictive performance of four survival models—Cox PH, RSF, DeepSurv, and DeepHit—in both time-dependent and non-time-dependent scenarios.

Simulation results demonstrated that when the PH assumption was satisfied, the Cox PH model slightly outperformed RSF, DeepSurv, and DeepHit in predictive accuracy. This advantage can be attributed to its simplicity and well-matched assumptions, which enable it to make more reliable predictions compared to more flexible but complex models. However, in real data analyses where the PH assumption was violated, machine learning and deep learning-based survival models demonstrated superior predictive performance. By dividing time into finer intervals, we were able to more precisely account for time-dependent coefficients and covariates, leading to improved predictive accuracy in both simulation and real-data scenarios. Using these refined intervals, we also proposed a novel method to compute an overall hazard ratio for each variant by weighting each time interval according to its duration relative to the total time covered by each COVID-19 variant.

Despite these contributions, our study has several limitations. First, there is insufficient evidence to definitively conclude that the Cox PH model performs worse than other survival models when the PH assumption is not met. Future research will involve additional simulations to assess whether machine learning and deep learning-based models consistently outperform the Cox PH model under these conditions. Additionally, our method of splitting intervals into equal-length segments when the PH assumption was violated may lead to segments with too few events, potentially affecting model stability. This highlights the need for improved strategies for defining time intervals. In subsequent studies, we will explore alternative methods to ensure a more balanced distribution of events within intervals and enhance the accuracy of time-dependent effect estimation. Furthermore, while we introduced a novel approach for integrating multiple time intervals in a stratified Cox PH model, we did not explicitly evaluate how well this method captures time-dependent effects. Future work will include simulations to validate its effectiveness in identifying such effects and refining the methodology accordingly.

## Conclusion

Our study presents a novel approach to modeling survival data with time-dependent effects by leveraging stratified Cox PH models and weighted hazard ratios across divided time intervals. Our findings demonstrate that while the Cox PH model performs well when its assumption holds, machine learning and deep learning models provide superior predictive accuracy when the PH assumption is violated. Although our method improves the estimation of time-dependent effects, further research is needed to refine the interval-splitting process, validate the proposed hazard ratio computation, and assess the effectiveness of our approach in capturing time-dependent effects. These future directions will contribute to more robust survival modeling techniques that better accommodate time-varying characteristics in real data.

## Supplementary Information

Supplementary Material 1. Note S1. Random survival forest (RSF). Note S2. DeepSurv. note S3. DeepHit. Figure S1. Boxplots for simulation results with time-dependent effects based on survival models and the number of time intervals. Figure S2. Boxplots for simulation results with time-dependent effects based on survival models and the event rate. Figure S3. Boxplots for simulation results with time-dependent effects based on survival models and the censoring rate. Figure S4. Boxplots for simulation results with time-dependent effects based on survival models and the SARS-CoV-2 infection rate. Table S1. The PH assumption test results for the Cox PH model with 10% event and no interval censoring. Table S2. All results from the simulation under the PH assumption. Table S3. The PH assumption test results for the Cox PH model with no time interval, 5% event and no interval censoring. Table S4. All results from the simulation with time-dependent effects. Table S5. The PH assumption test results for the stratified Cox PH models of all participants with 5 time-intervals. Table S6. The PH assumption test results for the stratified Cox PH models of all participants with 15 time-intervals. Table S7. All results from the survival analysis of all participants. Table S8. The PH assumption test results for the stratified Cox PH models with only SARS CoV-2-infected individuals. Table S9. All results from the survival analysis of only SARS-CoV-2-infected individuals with T2D or T2D PRS. Table S10. The hazard ratio and p-value of SARS-CoV-2 infection for each COVID-19 variant based on the stratified Cox PH model results with 15 time-intervals. Table S11. The hazard ratio and p-value of SARS-CoV-2 infection for five time-groups based on the stratified Cox PH model results with 5 time-intervals

## Data Availability

No datasets were generated or analysed during the current study.
