# Supplementary material for: Statistical learning methods for improving predictive performance in time-dependent survival models
Source: Genomics Inform. 2025 Sep 1;23:19. doi: 10.1186/s44342-025-00050-7 (PMC12400734; doi:10.1186/s44342-025-00050-7)
Supplement: Supplementary file 1 — Supplementary Material 1. Note S1. Random survival forest (RSF). Note S2. DeepSurv. note S3. DeepHit. Figure S1. Boxplots for simulation results with time-dependent effects based on survival models and the number of time intervals. Figure S2. Boxplots for simulation results with time-dependent effects based on survival models and the event rate. Figure S3. Boxplots for simulation results with time-dependent effects based on survival models and the censoring rate. Figure S4. Boxplots for simulation results with time-dependent effects based on survival models and the SARS-CoV-2 infection rate. Table S1. The PH assumption test results for the Cox PH model with 10% event and no interval censoring. Table S2. All results from the simulation under the PH assumption. Table S3. The PH assumption test results for the Cox PH model with no time interval, 5% event and no interval censoring. Table S4. All results from the simulation with time-dependent effects. Table S5. The PH assumption test results for the stratified Cox PH models of all participants with 5 time-intervals. Table S6. The PH assumption test results for the stratified Cox PH models of all participants with 15 time-intervals. Table S7. All results from the survival analysis of all participants. Table S8. The PH assumption test results for the stratified Cox PH models with only SARS CoV-2-infected individuals. Table S9. All results from the survival analysis of only SARS-CoV-2-infected individuals with T2D or T2D PRS. Table S10. The hazard ratio and p-value of SARS-CoV-2 infection for each COVID-19 variant based on the stratified Cox PH model results with 15 time-intervals. Table S11. The hazard ratio and p-value of SARS-CoV-2 infection for five time-groups based on the stratified Cox PH model results with 5 time-intervals [file 44342_2025_50_MOESM1_ESM.pdf]

Supplementary Materials to

Statistical Learning Methods for Improving Predictive Performance in Time-Dependent Survival Models

Hyungwoo Seo[1] and Wonil Chung[1,2]

Supplementary Notes

Note S1. Random survival forest (RSF)

The RSF is a non-parametric ensemble learning method used for survival analysis. It extends the concept of traditional decision trees to handle censored data, making it well-suited for survival scenarios where some event times are not fully observed. RSF constructs multiple survival trees using bootstrap samples from the original dataset, estimating the cumulative hazard function by aggregating the results from these individual trees. Each survival tree in RSF is constructed using a splitting rule that identifies the variable and split point to maximize survival differences, often based on the highest log-rank test value. This process is repeated iteratively until each terminal node has at least $d_0 > 0$ death events, ensuring that meaningful survival information remains in each node. For a given tree, the observed survival times in a terminal node h of the b-th bootstrap sample are represented as $\tilde{T}_{1,h} < \tilde{T}_{2,h} < \tilde{T}_{N(h),h}$. The cumulative hazard function for a terminal node is estimated with Nelson-Aalen estimator, and it is applied uniformly across individuals within the node:

$$\widehat{H}_h(t) = \sum_{t_{s,h} \leq t} \frac{d_{s,h}}{\tilde{Y}_{s,h}}$$

Here, $d_{s,h}$ represents the number of deaths by time s, and $\tilde{Y}_{s,h}$ is the number at risk at time $t_{s,h}$. By aggregating the cumulative hazard estimates from all the trees, RSF provides an overall estimate of the survival function, allowing for more robust and flexible modeling of complex survival relationships, especially when dealing with non-linear effects and interactions between covariates.

Note S2. DeepSurv

DeepSurv is a deep feed-forward neural network designed to predict the effects of covariates on an individual's hazard rate, which is parameterized by the weights of the network θ. The input to the network consists of baseline covariates x, which are passed through fully-connected layers, followed by a dropout layer to prevent overfitting. The network's output layer uses a linear activation function to estimate the log-risk, similar to that of the Cox PH model. The hazard function in DeepSurv is given by:

$$h(t|X_i) = h_0(t)e^{h_\theta(x)}$$

where $h_\theta(x)$ is the output of the neural network for the input covariates x. The baseline hazard function $h_0(t)$ is unspecified, similar to Cox PH model, allowing the network to learn the impact of covariates on the hazard rate.

The training process minimizes the average negative log partial likelihood and incorporates an additional regularization term to prevent overfitting. The objective function is given by:

$$\ell(\theta) := -\frac{1}{N_{E=1}} \sum_{i:E_i=1} \left(\hat{h}_\theta(x_i) - log \sum_{j \in \mathcal{R}(T_i)} e^{\hat{h}_\theta(x_j)} \right) + \lambda \times ||\theta||_2^2$$

Here, $N_{E=1}$ denotes the number of patients who experienced the event, and λ represents the ℓ_2 regularization (Ridge) parameter. DeepSurv uses gradient descent to minimize the negative log partial likelihood.

Note S3. DeepHit

DeepHit is a deep feed-forward neural network designed to predict the effects of covariates on an individual's hazard rate, and it is particularly adept at handling competing risks. Meaning it can simultaneously model the probabilities of different types of events occurring (e.g., death due to different causes). Unlike traditional survival models, such as the Cox PH model, DeepHit does not rely on a predefined parametric form of the hazard function. Instead, it utilizes a deep neural network to directly learn the joint distribution of survival times and event probabilities. The loss function in Deephit is represented as the sum of two terms: $\mathcal{L}_{total} = \mathcal{L}_1 + \mathcal{L}_2$ Here, $\mathcal{L}_1$ is the log-likelihood of the joint

distribution of the first hitting time and event, while $\mathcal{L}_2$ integrates a set of cause-specific ranking loss functions to improve predictive accuracy. The $\mathcal{L}_1$ term is defined to handle both uncensored and censored observations. The $\mathcal{L}_1$ term is given by:

$$\mathcal{L}_1 = -\sum_{i=1}^{N}\left[I\left(k^{(i)} \neq \emptyset\right)\cdot \log\left(y^{(i)}_{k^{(i)},s^{(i)}}\right) + I\left(k^{(i)} \neq \emptyset\right)\cdot\log\left(1-\sum_{k=1}^{K}\hat{F}_k\left(s^{(i)}\middle|x^{(i)}\right)\right)\right]$$

Here, $y_{k,s}$ represents the estimated probabilty that an event k occurs at time s. The function $F_k(s|x)$ is the cumulative incidence function that sums over the estimated probabilities of the event occurring up to time s. For censored observations, it accounts for the probability that no event has occurs up to the censoring time, thereby ensuring that the model appropriately handles incomplete data.

The $\mathcal{L}_2$ term is a ranking loss function that ensures proper ordering of survival risks. It is designed to assign higher risk scores to individuals who experienced an event earlier compared to those experiencing it later. The $\mathcal{L}_2$ term is given by:

$$\mathcal{L}_2 = \sum_{k=1}^{K}\alpha_k\cdot\sum_{i\neq j}A_{k,i,j}\cdot\eta\left(\hat{F}_k\left(s^{(i)}\middle|x^{(i)}\right),\hat{F}_k\left(s^{(i)}\middle|x^{(j)}\right)\right)$$

Here, α_k is a weight that controls the influence of the ranking loss for event k. $A_{k,i,j}$ is an indicator function that determines whether a pair of patients can be compared for event k. It is used to select only the pairs where one patient experienced the event earlier than the other. $\eta(x,y) = \exp\left(\frac{-(x-y)}{\sigma}\right)$ is a convex loss function that penalizes incorrect rankings, with σ being a scaing parameter.

Supplementary Figures

Figure S1. Boxplots for simulation results with time-dependent effects based on survival models and the number of time intervals.

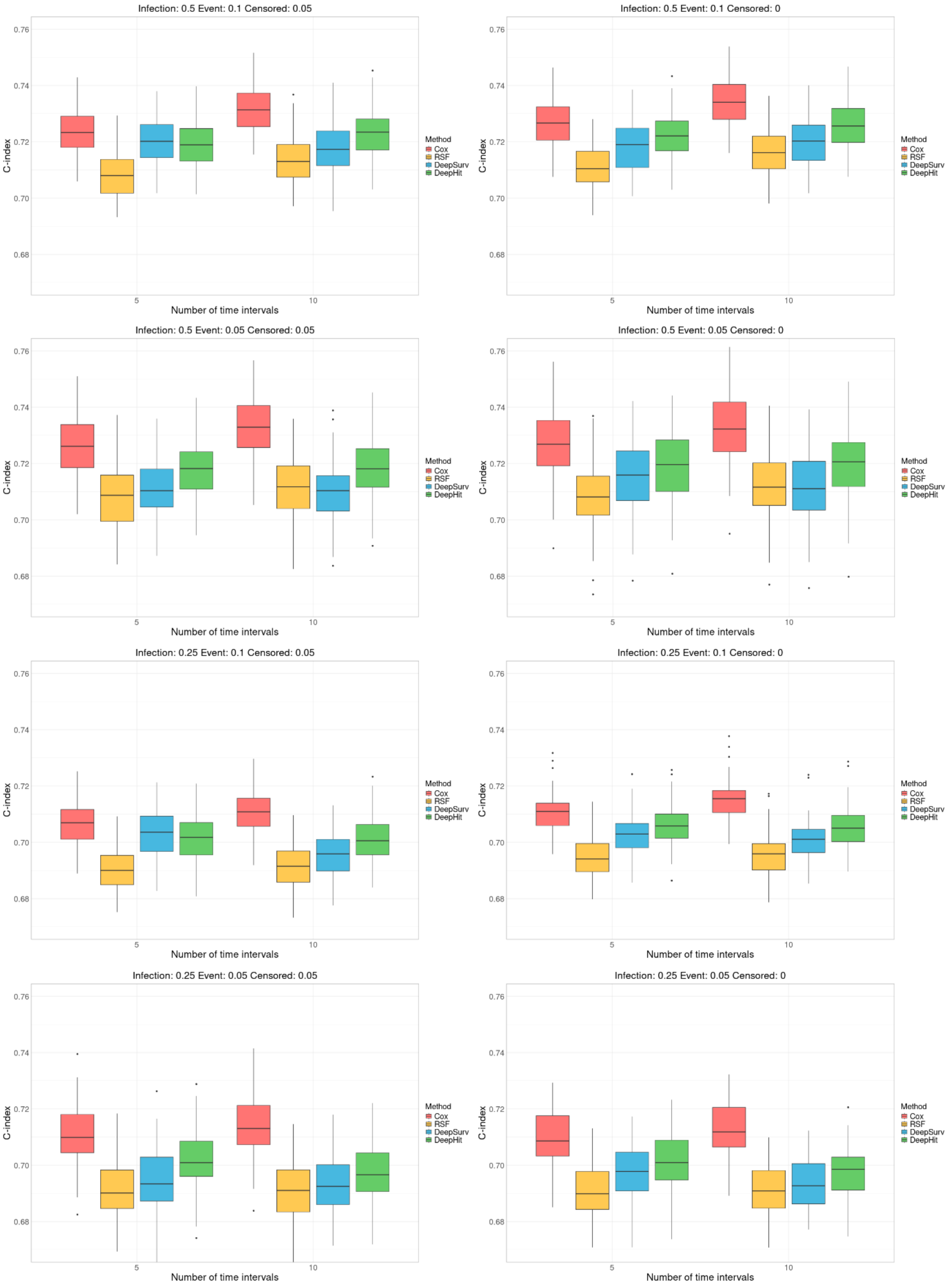

Figure S2. Boxplots for simulation results with time-dependent effects based on survival models and the event rate.

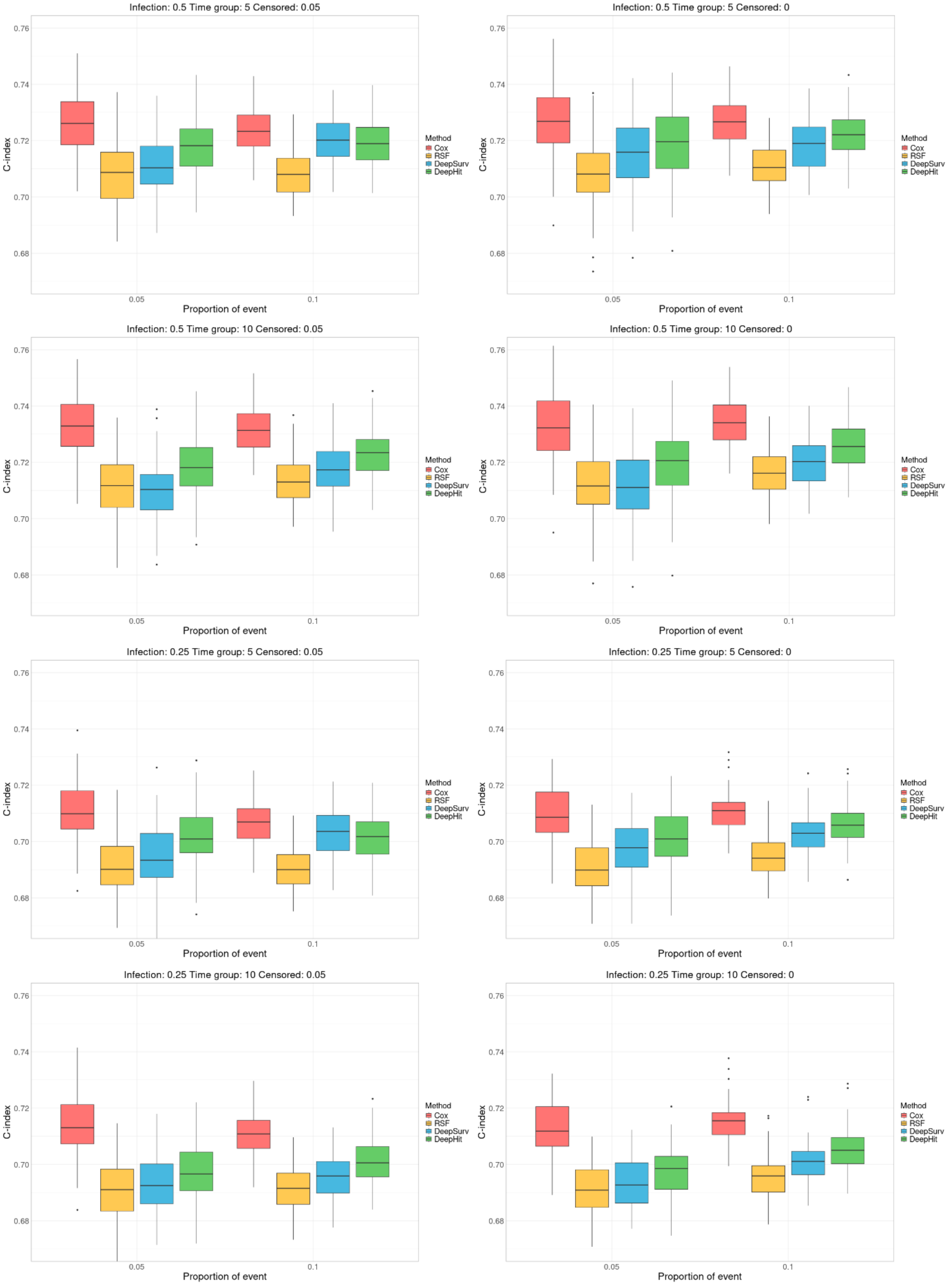

Figure S3. Boxplots for simulation results with time-dependent effects based on survival models and the censoring rate.

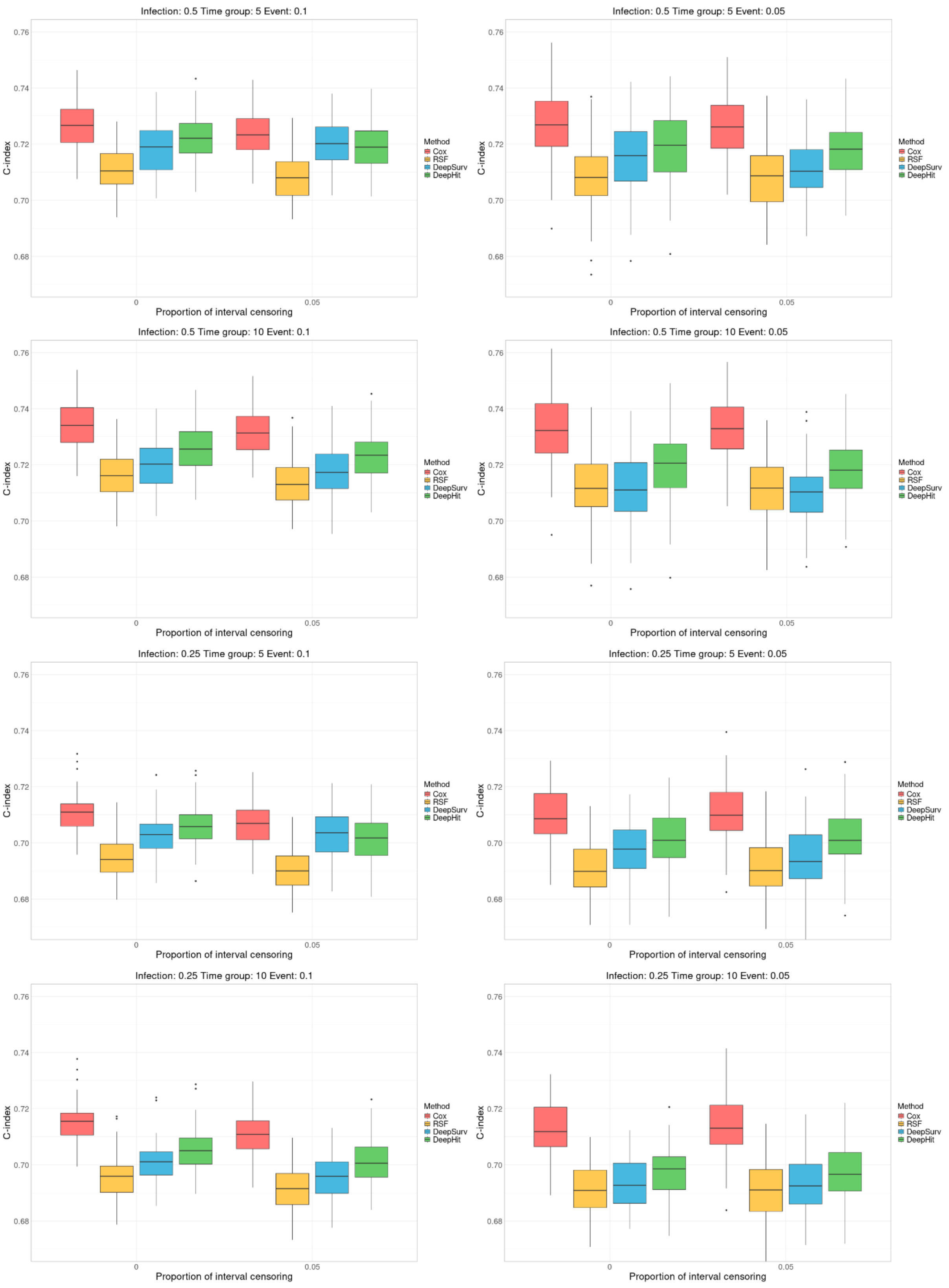

Figure S4. Boxplots for simulation results with time-dependent effects based on survival models and the SARS-CoV-2 infection rate.

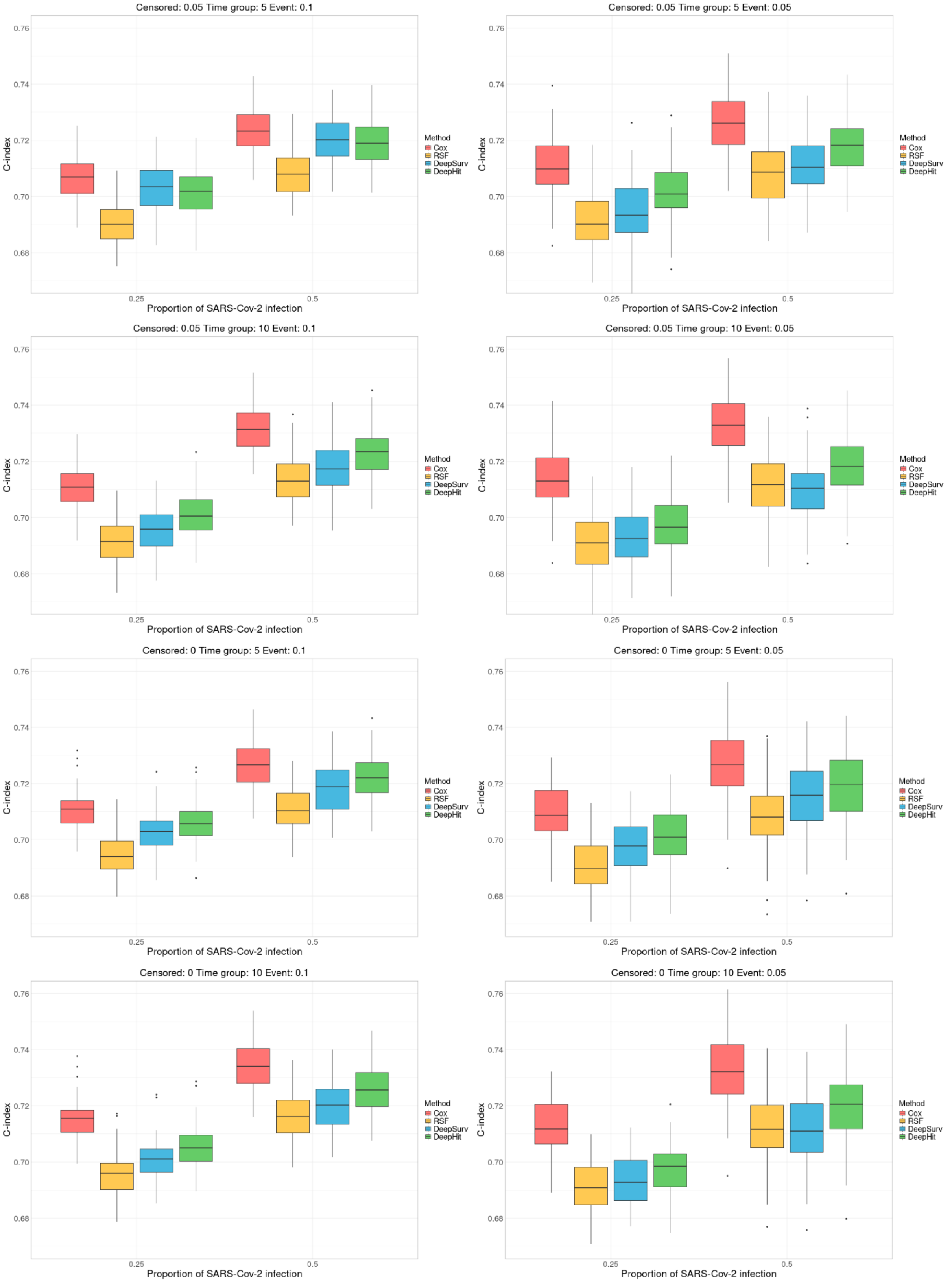

Supplementary Tables

Table S1. The PH assumption test results for the Cox PH model with 10% event and no interval censoring.

Covariates	χ^2	df	p-value
COVID-19 variant	6.2879	6	0.392
T2D	0.0219	1	0.882
Age	1.6682	1	0.197
Sex	1.8777	1	0.171
BMI	0.002	1	0.965
Genotype array	0.0974	1	0.755
GLOBAL	10.2849	11	0.505

Table S2. All results from the simulation under the PH assumption.

Event	Censored	CoxPH		RSF		DeepSurv		DeepHit	
		Mean	SD	Mean	SD	Mean	SD	Mean	SD
5%	0%	0.776	0.009	0.756	0.010	0.768	0.010	0.765	0.011
	5%	0.781	0.010	0.759	0.012	0.772	0.011	0.768	0.013
10%	0%	0.777	0.007	0.760	0.007	0.772	0.007	0.769	0.009
	5%	0.771	0.006	0.754	0.007	0.766	0.007	0.762	0.008

Clinical covariates : Age, Sex, BMI, Geno array, T2D, COVID-19 variants

Table S3. The PH assumption test results for the Cox PH model with no time interval, 5% event and no interval censoring.

Covariates	χ^2	df	p-value
SARS-CoV-2 infection	14.0767	1	0.0002
T2D	2.7232	1	0.0989
Age	2.4542	1	0.1172
Sex	0.7008	1	0.4025
BMI	1.4545	1	0.2278
Genotype array	0.0838	1	0.7722
GLOBAL	19.6489	6	0.0032

Table S4. All results from the simulation with time-dependent effects.

SARS-CoV-2 infection	Event	Censored	CoxPH		Stratified Cox PH 5 time intervals		Stratified Cox PH 10 time intervals		RSF 0 time intervals		RSF 5 time intervals		RSF 10 time intervals	
			Mean	SD	Mean	SD	Mean	SD	Mean	SD	Mean	SD	Mean	SD
50%	5%	0%	0.689	0.013	0.727	0.012	0.733	0.012	0.671	0.013	0.709	0.012	0.712	0.012
		5%	0.689	0.010	0.726	0.010	0.733	0.010	0.671	0.014	0.709	0.011	0.712	0.012
	10%	0%	0.687	0.009	0.726	0.008	0.734	0.008	0.678	0.010	0.711	0.008	0.716	0.008
		5%	0.684	0.008	0.724	0.008	0.732	0.008	0.676	0.010	0.710	0.008	0.715	0.008
25%	5%	0%	0.682	0.010	0.710	0.009	0.713	0.009	0.660	0.012	0.691	0.009	0.691	0.009
		5%	0.682	0.011	0.711	0.011	0.714	0.011	0.660	0.013	0.691	0.011	0.691	0.011
	10%	0%	0.681	0.007	0.711	0.007	0.715	0.007	0.669	0.009	0.694	0.007	0.695	0.007
		5%	0.679	0.009	0.707	0.008	0.711	0.008	0.667	0.009	0.691	0.008	0.691	0.008

SARS-CoV-2 infection	Event	Censored	DeepSurv 0 time intervals		DeepSurv 5 time intervals		DeepSurv 10 time intervals		DeepHit 0 time intervals		DeepHit 5 time intervals		DeepHit 10 time intervals	
			Mean	SD	Mean	SD	Mean	SD	Mean	SD	Mean	SD	Mean	SD
50%	5%	0%	0.706	0.012	0.715	0.013	0.712	0.013	0.702	0.013	0.719	0.012	0.720	0.013
		5%	0.704	0.011	0.711	0.010	0.710	0.010	0.700	0.016	0.719	0.010	0.719	0.011
	10%	0%	0.705	0.008	0.718	0.009	0.720	0.009	0.703	0.010	0.722	0.008	0.726	0.008
		5%	0.702	0.009	0.720	0.008	0.720	0.008	0.700	0.010	0.721	0.008	0.725	0.008
25%	5%	0%	0.696	0.010	0.698	0.009	0.693	0.009	0.691	0.017	0.701	0.009	0.697	0.009
		5%	0.695	0.012	0.695	0.011	0.693	0.011	0.692	0.013	0.701	0.011	0.697	0.010
	10%	0%	0.697	0.007	0.703	0.007	0.700	0.007	0.695	0.007	0.706	0.007	0.705	0.007
		5%	0.693	0.009	0.703	0.009	0.696	0.008	0.693	0.008	0.702	0.009	0.701	0.009

Clinical Covariates: Age, Sex, BMI, Genotype array, T2D, SARS-CoV-2

Table S5. The PH assumption test results for the stratified Cox PH models of all participants with 5 time-intervals.

Covariates	$\chi 2$	df	p-value
SARS-CoV-2 infection in time-group 1	1.746	1	0.1863
SARS-CoV-2 infection in time-group 2	2.665	1	0.1026
SARS-CoV-2 infection in time-group 3	3.138	1	0.0765
SARS-CoV-2 infection in time-group 4	3.704	1	0.0543
SARS-CoV-2 infection in time-group 5	0.133	1	0.7152
T2D	0.229	1	0.6324
Age	0.071	1	0.7900
Sex	0.314	1	0.5752
BMI	2.949	1	0.0859
Genotype array	0.529	1	0.4670
PC 1	< 0.001	1	0.9983
PC 2	6.115	1	0.0134
PC 3	0.019	1	0.8894
PC 4	4.616	1	0.0317
GLOBAL	24.921	14	0.0354

Table S6. The PH assumption test results for the stratified Cox PH models of all participants with 15 time-intervals.

Covariates	χ2	df	p-value
SARS-CoV-2 infection in time-group 1	0.838	1	0.3599
SARS-CoV-2 infection in time-group 2	0.003	1	0.9567
SARS-CoV-2 infection in time-group 3	0.691	1	0.4058
SARS-CoV-2 infection in time-group 4	1.809	1	0.1786
SARS-CoV-2 infection in time-group 5	1.424	1	0.2328
SARS-CoV-2 infection in time-group 6	0.25	1	0.6172
SARS-CoV-2 infection in time-group 7	0.066	1	0.7972
SARS-CoV-2 infection in time-group 8	0.194	1	0.6596
SARS-CoV-2 infection in time-group 9	1.75	1	0.1859
SARS-CoV-2 infection in time-group 10	0.105	1	0.7454
SARS-CoV-2 infection in time-group 11	0.009	1	0.9255
SARS-CoV-2 infection in time-group 12	2.662	1	0.1027
SARS-CoV-2 infection in time-group 13	3.137	1	0.0765
SARS-CoV-2 infection in time-group 14	3.705	1	0.0542
SARS-CoV-2 infection in time-group 15	0.133	1	0.7150
T2D	2.637	1	0.1044
Age	2.195	1	0.1385
Sex	0.019	1	0.8913
BMI	5.787	1	0.0161
Genotype array	0.665	1	0.4149
PC 1	0.013	1	0.9095
PC 2	7.502	1	0.0062
PC 3	0.03	1	0.8617
PC 4	4.751	1	0.0293
GLOBAL	36.745	24	0.0464

Table S7. All results from the survival analysis of all participants.

Model	CoxPH				RSF						Deephit					
Number of time groups	5		15		0		5		15		0		5		15	
	mean	sd	mean	sd	mean	sd	mean	sd	mean	sd	mean	sd	mean	sd	mean	sd
T2D+Clinical	0.764	0.005	0.769	0.005	0.755	0.007	0.763	0.005	0.760	0.004	0.753	0.005	0.765	0.005	0.769	0.005
T2D PRS+ Clinical	0.755	0.005	0.760	0.005	0.755	0.007	0.753	0.005	0.751	0.004	0.742	0.006	0.755	0.005	0.759	0.005

Clinical covariates: SARS-CoV-2, Age, Sex, BMI, Geno array, Top 4 PCs

Table S8. The PH assumption test results for the stratified Cox PH models with only SARS-CoV-2-infected individuals.

**Covariates**	**$\chi 2$**	**df**	**P-value**
COVID-19 variants	287.753	6	3.44E-59
T2D	9.114	1	2.54E-03
Age	1.126	1	2.89E-01
Sex	12.091	1	5.07E-04
BMI	14.359	1	1.51E-04
Genotype array	0.546	1	4.60E-01
PC 1	0.908	1	3.41E-01
PC 2	1.558	1	2.12E-01
PC 3	0.224	1	6.36E-01
PC 4	2.924	1	8.73E-02
GLOBAL	312.741	15	1.25E-57

Table S9. All results from the survival analysis of only SARS-CoV-2-infected individuals with T2D or T2D PRS.

	**CoxPH**		**RSF**		**Deepsurv**		**Deephit**	
**Model**	**Mean**	**SD**	**Mean**	**SD**	**Mean**	**SD**	**Mean**	**SD**
T2D + Clinical	0.796	0.077	0.807	0.013	0.817	0.012	0.821	0.012
T2D + COVID-19 variants + Clinical	0.812	0.073	0.857	0.014	0.820	0.086	0.859	0.014
T2D PRS + Clinical	0.789	0.075	0.795	0.013	0.808	0.011	0.819	0.013
T2D PRS + COVID-19 variants + Clinical	0.808	0.071	0.850	0.015	0.818	0.077	0.854	0.014

Clinical covariates: Age, Sex, BMI, Geno array, Top 4 PCs

Table S10. The hazard ratio and p-value of SARS-CoV-2 infection for each COVID-19 variant based on the stratified Cox PH model results with 15 time-intervals.

**Covariates**	**HR (95% CI)**	***p*-value**
	early: 29.359 (25.123–34.308)	$< 2.23 \times 10^{-308}$
	eu1: 20.734 (18.841–22.817)	$< 2.23 \times 10^{-308}$
	alpha: 4.079 (3.486–4.773)	8.50×10^{-69}
SARS-CoV-2 + Clinical	delta: 2.057 (1.850–2.286)	3.81×10^{-38}
	omicron1: 1.883 (1.653–2.145)	9.76×10^{-21}
	omicron2: 1.924 (1.708–2.166)	2.57×10^{-26}
	omicron3: 2.006 (1.761–2.285)	6.49×10^{-25}

Clinical covariates: T2D, Age, Sex, BMI, Geno array, Top 4 PCs

Table S11. The hazard ratio and p-value of SARS-CoV-2 infection for five time-groups based on the stratified Cox PH model results with 5 time-intervals.

**Covariates**	**HR (95% CI)**	***p*-value**
	before delta: 7.333 (6.902 - 7.791)	$< 2.23 \times 10^{-308}$
	delta: 2.013 (1.811 - 2.238)	2.61×10^{-38}
SARS-CoV-2 + Clinical	omicron1: 1.864 (1.636 - 2.123)	7.54×10^{-21}
	omicron2: 1.906 (1.692 - 2.146)	1.82×10^{-26}
	omicron3: 1.986 (1.744 - 2.262)	4.83×10^{-25}

Clinical covariates: T2D, Age, Sex, BMI, Geno array, Top 4 PCs
